# The Protective Role of Intermedin in Contrast-Induced Acute Kidney Injury: Enhancing Peritubular Capillary Endothelial Cell Adhesion and Integrity Through the cAMP/Rac1 Pathway

**DOI:** 10.3390/ijms252011110

**Published:** 2024-10-16

**Authors:** Tingting Gao, Ruiyuan Gu, Heng Wang, Lizheng Li, Bojin Zhang, Jie Hu, Qinqin Tian, Runze Chang, Ruijing Zhang, Guoping Zheng, Honglin Dong

**Affiliations:** 1 The Second Clinical Medical College, Shanxi Medical University, Taiyuan 030000, China; gaotingting@sxmu.edu.cn (T.G.); gry1997@outlook.com (R.G.); wangheng7015@mails.jlu.edu.cn (H.W.); lilizheng@sxmu.edu.cn (L.L.); hujie_2001@126.com (J.H.); tianqinqin_1225@163.com (Q.T.); changrunze1@sxmu.edu.cn (R.C.); zhangruijing@sxmu.edu.cn (R.Z.); 2 Centre for Transplant and Renal Research, Westmead Institute for Medical Research, The University of Sydney, Sydney 201101, Australia; 3 Department of Biochemistry and Molecular Biology, College of Basic Medical Sciences, Shanxi Medical University, Taiyuan 030000, China; zhangboj90@126.com

**Keywords:** contrast-induced acute kidney injury, contrast media, intermedin, peritubular capillaries, endothelial barrier, cAMP, Rac1

## Abstract

Contrast-induced acute kidney injury (CIAKI) is a common complication with limited treatments. Intermedin (IMD), a peptide belonging to the calcitonin gene-related peptide family, promotes vasodilation and endothelial stability, but its role in mitigating CIAKI remains unexplored. This study investigates the protective effects of IMD in CIAKI, focusing on its mechanisms, particularly the cAMP/Rac1 signaling pathway. Human umbilical vein endothelial cells (HUVECs) were treated with iohexol to simulate kidney injury in vitro. The protective effects of IMD were assessed using CCK8 assay, flow cytometry, ELISA, and Western blotting. A CIAKI rat model was utilized to evaluate renal peritubular capillary endothelial cell injury and renal function through histopathology, immunohistochemistry, immunofluorescence, Western blotting, and transmission electron microscopy. In vitro, IMD significantly enhanced HUVEC viability and mitigated iohexol-induced toxicity by preserving intercellular adhesion junctions and activating the cAMP/Rac1 pathway, with Rac1 inhibition attenuating these protective effects. In vivo, CIAKI caused severe damage to peritubular capillary endothelial cell junctions, impairing renal function. IMD treatment markedly improved renal function, an effect negated by Rac1 inhibition. IMD protects against renal injury in CIAKI by activating the cAMP/Rac1 pathway, preserving peritubular capillary endothelial integrity and alleviating acute renal injury from contrast media. These findings suggest that IMD has therapeutic potential in CIAKI and highlight the cAMP/Rac1 pathway as a promising target for preventing contrast-induced acute kidney injury in at-risk patients, ultimately improving clinical outcomes.

## 1. Introduction

In recent years, with the rapid development of radiological diagnosis and angiography, iodized contrast media have become the most widely used reagent in diagnostic angiography and catheter interventional therapy in patients with cardiovascular disease (CVD) [1]. The incidence of CIAKI has concurrently surged, becoming the third most prevalent cause of acute renal injury among hospitalized patients [2]. CIAKI, resulting from the intravascular administration of iodine-based contrast media (CM), is a significant complication in this context [3]. To date, the pathogenesis of CIAKI has not been completely clarified.

Previous studies on CIAKI have predominantly focused on the mechanisms by which CM induce damage to renal tubules and tubular epithelial cells. However, research investigating its impact on peritubular capillaries (PTCs) remains limited [4,5,6]. Studies have revealed a significant increase in the diameter of PTCs following intravenous injection of CM in mice, indicating that PTCs play a crucial role in the onset and progression of CIAKI [7,8]. PTCs are secondary capillaries in the kidney, densely distributed around the renal tubules [9]. They serve as the primary nourishing blood vessels for both the renal tubules and the renal interstitium, with their function being highly dependent on the integrity of the vascular endothelial barrier [10]. Following the intravascular injection of CM, renal peritubular endothelial cells (RPECs) are directly exposed to CM within the plasma. This exposure can trigger inflammation, elevate the production of reactive oxygen species (ROS) in endothelial cells, increase plasma osmotic pressure, and induce hemodynamic changes, all contributing to RPEC injury [11]. Some researchers have suggested that in acute renal failure (ARF), the disruption of the endothelial barrier function in RPECs can cause PTC leakage, reducing perivascular perfusion and impairing the delivery of oxygen and nutrients to renal tubular epithelial cells, thereby compromising renal function [11,12]. The endothelial barrier primarily consists of adherens junctions (AJs), tight junctions (TJs), and the cytoskeleton [13,14]. Among these, AJs are more widely distributed and have a stronger influence on microvascular function. AJs are primarily composed of vascular endothelial cadherin (VE-cadherin), catenins, and actin [15]. GTPases of the Rho family, including RhoA, Rac1, and Cdc42, are widely recognized as critical regulators of the actin cytoskeleton and AJs, playing a pivotal role in maintaining the integrity of the endothelial barrier [16,17].

Based on the critical role of endothelial integrity in the pathogenesis of CIAKI, identifying effective therapeutic agents is crucial. One promising candidate is IMD, a member of the calcitonin gene-related peptide (CGRP) superfamily, which is expressed in various endothelial cells [18]. IMD interacts with the cell membrane receptor complex CRLR/RAMPs, participating in the regulation of numerous physiological and pathological processes, including vasodilation, stabilization of the endothelial barrier, and inhibition of apoptosis [19,20,21]. Studies have demonstrated that in endothelial cells, IMD binding to its receptor activates adenylate cyclase, resulting in increased intracellular cAMP levels [22,23]. This rise in cAMP can subsequently activate Rac1, which plays a crucial role in protecting endothelial cells and maintaining the integrity of the endothelial barrier [13]. In CIAKI, damage to PTCs can impair renal tubular function [24]. Consequently, it is hypothesized that PTC damage significantly contributes to renal impairment in CIAKI, and that IMD may exert renoprotective effects by preserving PTC integrity through the cAMP/Rac1 signaling pathway. Notably, IMD exhibits varying effects on the endothelial barrier in different vascular beds. For instance, in isolated rat lungs and human lung endothelial cells, IMD has been shown to stabilize the endothelial barrier via the cAMP/protein kinase A (PKA) pathway [25,26]. Conversely, in the rat coronary microvascular system, IMD mediates cAMP activation while inactivating both RhoA/Rock and Rac1 signaling pathways in coronary microvascular endothelial cells. Disruption of the actin cytoskeleton leads to the loss of microvascular endothelial barrier in the coronary artery [27]. However, the pathological and physiological effects of IMD on the vascular barrier of PTCs in CIAKI remain largely unknown. It is still unclear whether IMD can mitigate the endothelial barrier disruption induced by CM in the CIAKI model and what specific mechanisms are involved in this process.

This study aimed to elucidate the potential of IMD as a therapeutic agent in CIAKI by investigating its role in preserving PTC integrity through the cAMP/Rac1 signaling pathway. Specifically, we aimed to determine whether IMD could mitigate endothelial barrier disruption induced by CM and to explore the underlying mechanisms involved.

## 2. Results 

### 2.1. IMD Mitigated Iohexol-Induced Damage to HUVEC Viability and Apoptosis 

**Figure 1:**
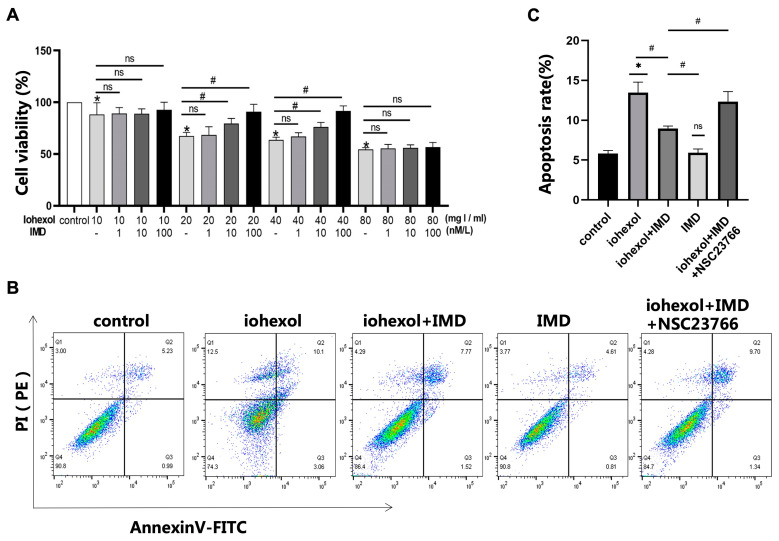
IMD can antagonize damage to HUVEC viability and apoptosis induced by iohexol. (**A**) HUVECs were preincubated with IMD (0, 1, 10, 100 nmol/L) for 30 min and then treated with iohexol (10, 20, 40, 80 mgI/mL) for 12 h. A CCK-8 kit was used to test cell viability. (**B**) HUVECs were preincubated with IMD (10 nmol/L) for 30 min, then were treated with iohexol (40 mgI/mL) or iohexol (40 mgI/mL) +NSC23766(50 μM) for 12 h. Apoptosis was detected using flow cytometry. (**C**) The apoptosis rate in each group was quantified (*n* = 6). The results were analyzed using ANOVA, followed by Tukey’s multiple comparisons test for subgroup analysis. All data are expressed as mean ± SD, * *p* < 0.05 compared to the control group, # *p* < 0.05. For comparison between groups, ns: not significant.

CCK8 assays demonstrated that iohexol reduced HUVEC activity in a dose-dependent manner. However, IMD significantly alleviated this reduction, thereby preserving HUVEC viability (Figure 1A). Flow cytometry analysis revealed that iohexol substantially increased HUVEC apoptosis, which IMD effectively inhibited. The percentages of apoptotic cells (Q2 + Q3) in the control, iohexol, iohexol + IMD, IMD, and iohexol + IMD + NSC23766 groups were 6.22%, 13.16%, 9.29%, 5.42%, and 11.04%, respectively (Figure 1B, *p* < 0.05). The quantification of apoptosis results is presented in Figure 1C.

### 2.2. IMD Protected the Adherent Junctions in HUVECs by Activating the cAMP/Rac1 Pathway

**Figure 2:**
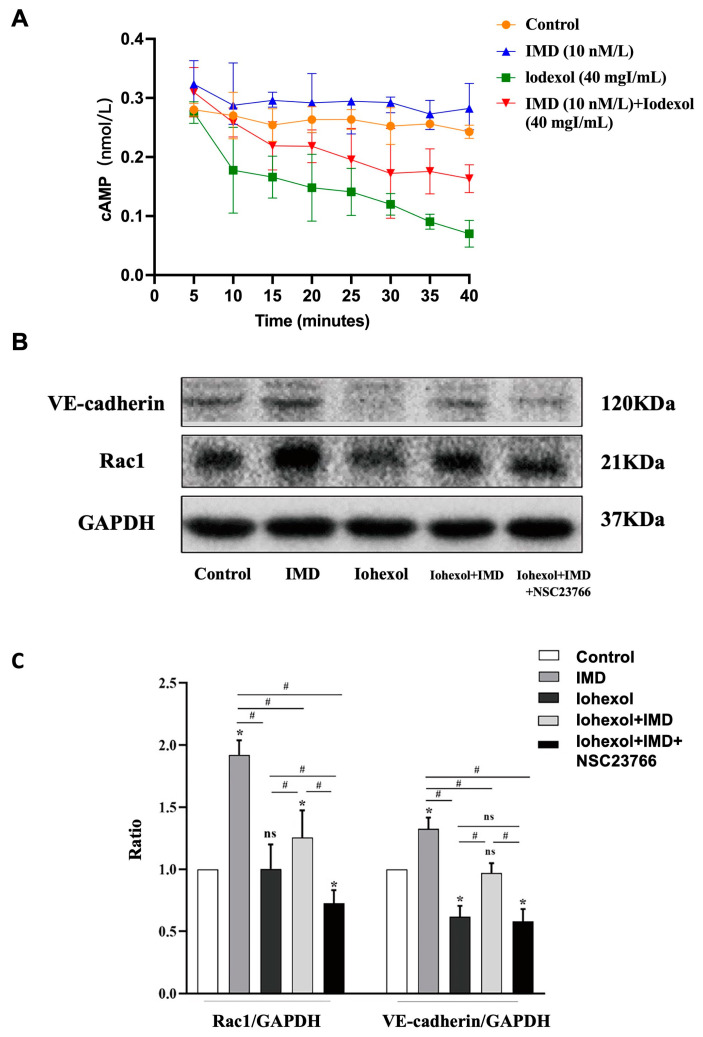
IMD can protect the adherens junction of HUVECs by activating the cAMP/Rac1 pathway. (**A**) Intermedin induces cAMP production in HUVECs. The concentration of cAMP was measured using ELISA, *n* = 4. (**B**,**C**) HUVECs were treated with 10 nmol of IMD and/or 40 mgI/mL iohexol or 50 μM NSC23766 for 12 h. Whole-cell lysates of HUVECs were collected for immunoblotting analysis of Rac1, VE-cadherin, and GAPDH. The results were analyzed using ANOVA, with Tukey’s multiple comparisons test applied for subgroup analysis. All data are expressed as mean ± SD, * *p* < 0.05 compared to the control group, ^#^ *p* < 0.05. For comparison between groups, ns: not significant.

ELISA assays measuring cAMP levels indicated that, compared to basal levels in the control group, the cAMP concentration rapidly and significantly increased within 5 min of exposure to IMD (10 nmol/L) (Figure 2A). Western blot analysis was employed to detect the expression levels of Rac1 and VE-cadherin in groups treated with IMD, iohexol + IMD, and iohexol + IMD + NSC23766 (a specific Rac1 inhibitor [28]) (Figure 2B). Compared with the iohexol group, the expression of Rac1 and VE-cadherin were elevated in the iohexol + IMD treatment group, but the improvement effect of IMD disappeared after Rac1 was inhibited (Figure 2C). These findings suggest that IMD activates the cAMP/Rac1 pathway and protects the adherens junctions between HUVECs, while the application of the Rac1 inhibitor significantly diminishes this protective effect.

### 2.3. IMD Attenuated Renal Injury in Rat CIAKI Models and Inhibition of Rac1 Negated This Protective Effect

**Figure 3:**
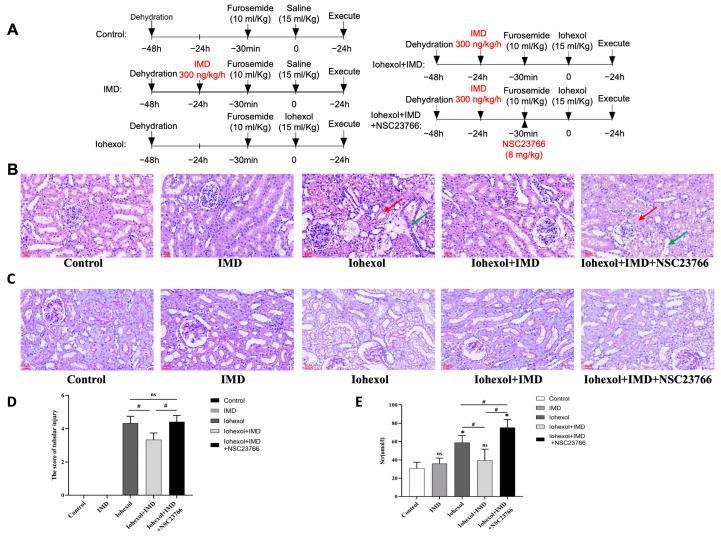
IMD attenuates renal injury in rat CIAKI models, and inhibition of Rac1 might abolish this protective effect. (**A**) Male SD rats were randomly divided into five groups (*n* = 6), and the rats in each group were treated as shown in the scheme. (**B**) Renal morphology (HE, magnification ×400) showed vacuolar degeneration of renal tubules (shown by red arrows) and dilatation of renal tubules (shown by green arrows). (**C**) Magnification of renal PAS staining (×400) showed the absence of brush edges of renal tubules, vacuolar degeneration, and dilatation of renal tubules. (**D**) Renal tubular injury score. (**E**) Serum creatinine levels for the indicated treatments. The results were analyzed using ANOVA, with Tukey’s multiple comparisons test applied for subgroup analysis. All data are expressed as mean ± SD, * *p* < 0.05 compared to the control group, # *p* < 0.05. For comparison between groups, ns: not significant.

SD rats were randomly divided into five groups (*n* = 6): a control group, an IMD group, an iohexol group, an iohexol + IMD group, and an iohexol + IMD + NSC23766 group. The treatment protocol for each group is illustrated in Figure 3A. Serum creatinine levels, a marker of renal function, were measured. Kidney sections from the rats were stained with HE (Figure 3B) and PAS (Figure 3C), and the extent of renal tubular injury was assessed through pathological scoring (Figure 3D). The HE staining results indicated that the iohexol group exhibited significant tubular vacuolar degeneration and tubular dilation compared to the control group. Notably, the addition of IMD resulted in a marked improvement in pathological damage. However, when Rac1 was inhibited, the protective effects of IMD were abrogated. Serum creatinine levels in the iohexol group were significantly elevated compared to the control group. However, in the IMD + iohexol group, serum creatinine levels were notably reduced compared to the iohexol group, indicating that IMD mitigates iohexol-induced renal dysfunction. Conversely, the addition of NSC23766 led to a significant increase in serum creatinine, implying that the protective effect of IMD was nullified by Rac1 inhibition (Figure 3E). Moreover, the pathological score for renal tubular injury in the IMD + iohexol group was significantly lower than in the iohexol group and the IMD + iohexol + NSC23766 group. These findings suggest that IMD attenuates renal injury in CIAKI rats, and Rac1 inhibition negates this protective effect, indicating that IMD may alleviate CIAKI in rats via the cAMP/Rac1 pathway.

### 2.4. IMD Activated the cAMP/Rac1 Pathway and Mitigated Peritubular Capillary Injury in CIAKI Rats

**Figure 4:**
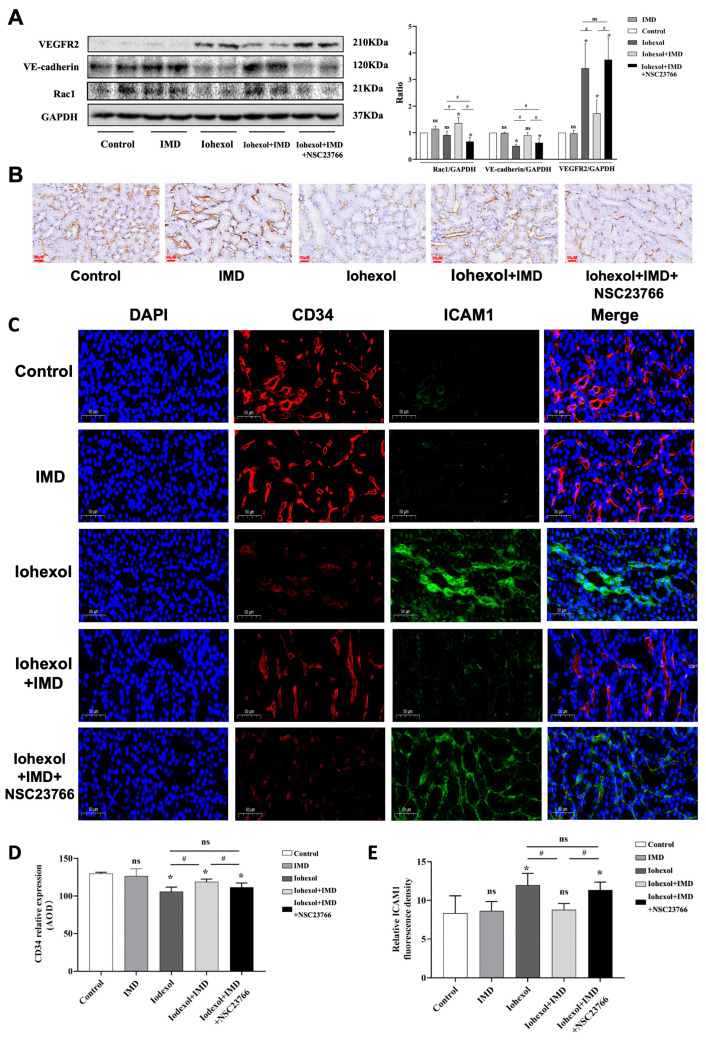
IMD activates the cAMP/Rac1 pathway and alleviates renal peritubular capillary injury in CIAKI rats. (**A**,**B**): The renal lysates of rats were collected and the expression of Rac1, VE-cadherin, VEGFR2, and GAPDH were detected using Western blotting. (**C**) Kidney sections were stained with CD34 immunohistochemical staining (magnification, ×400, scale 30 μm). (**D**) Kidney sections were stained with CD34 and ICAM1 immunofluorescence staining (magnification, ×200, scale 50 μm). (**E**) The average optical density of CD34 positive staining in each group was quantified (*n* = 6). (**E**) Quantification of the average optical density of ICAM1 immunofluorescence (*n* = 6). The data were analyzed using ANOVA, followed by Tukey’s multiple comparisons test for subgroup analysis. All data are expressed as mean ± SD, * *p* < 0.05 compared to the control group, # *p* < 0.05. For comparison between groups, ns: not significant.

Western blot analysis was conducted to examine the levels of Rac1, VEGFR2 and VE-cadherin, focusing on the cAMP/Rac1 and adhesion pathways in IMD-mediated effects within the CIAKI rat model. IMD enhanced the expression of Rac1 and VE-cadherin; however, these effects were nullified by the specific inhibition of Rac1. This suggests that IMD mitigates iohexol-induced destruction of adherens junctions through activation of the cAMP/Rac1 pathway. Additionally, it was observed that iohexol stimulation elevated VEGFR2 expression, a critical receptor for neovascularization, whereas IMD attenuated this increase, and specific Rac1 inhibition also blocked the IMD-induced effects [29,30]. This indicates that iohexol inflicts damage on renal microvessels, leading to a reactive increase in VEGFR2 expression, which promotes neovascularization. Our findings demonstrate that IMD protects renal microvessels from iohexol-induced damage, and this protective effect is inhibited by Rac1 blockade (Figure 4A,B), further supporting the involvement of the cAMP/Rac1 pathway in the action of IMD. Relative expressions of CD34 and ICAM1 were evaluated using immunohistochemistry and immunofluorescence to assess peritubular capillary damage. As a marker of microvascular endothelium, CD34 expression inversely correlates with the extent of microvascular damage [31]. Results revealed that CD34 expression was significantly lower in the CIAKI group compared to the control group, while the IMD + iohexol group exhibited significantly higher CD34 levels compared to the CIAKI group, with NSC23766 reversing effects of IMD (Figure 4C–E). ICAM-1, also known as CD54, is part of the immunoglobulin superfamily of adhesion molecules, and its increased expression indicates vascular endothelial injury [32]. In CIAKI rat kidneys, ICAM1 expression was elevated, but IMD intervention significantly reduced ICAM1 levels, an effect that was abolished by Rac1 inhibition. These findings suggest that IMD mitigates renal microvascular injury in CIAKI rats by activating the cAMP/Rac1 pathway.

### 2.5. IMD Can Protect the Endothelial Barrier of PTCs

**Figure 5:**
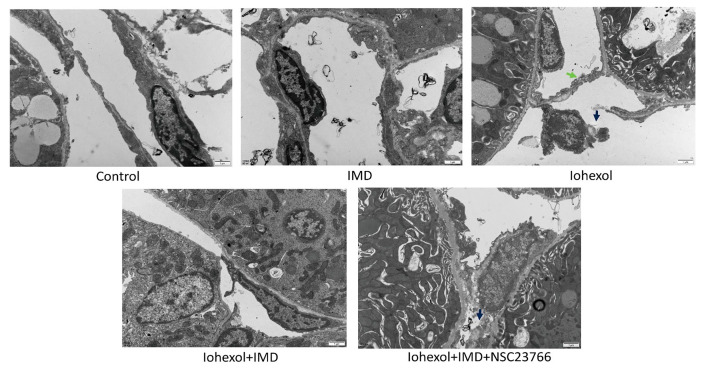
IMD protects the PTC endothelial barrier: a representative microphotograph of the ultrastructural changes of peritubular capillary endothelial cells, cell membrane discontinuity (shown by green arrows), and basement membrane fracture (shown by blue arrows) (original magnification, ×12,000; scale, 1 μm).

The ultrastructure of the rat kidney was examined using transmission electron microscopy (Figure 5). The results showed that contrast media (CM) led to vascular endothelial cell necrosis, disrupted cell membranes, and basement membrane rupture, whereas IMD significantly mitigated damage to the PTC endothelial barrier. However, this protective effect was abolished upon Rac1 inhibition. These findings further support that IMD protects the PTC endothelial barrier in CIAKI rats through the cAMP/Rac1 pathway.

## 3. Discussion

This study demonstrates that exogenous IMD significantly protects against contrast media-induced endothelial injury in a rat model of CIAKI. Specifically, IMD reduces PTC damage and overall cellular injury by activating the cAMP/Rac1 signaling pathway, which helps maintain endothelial barrier integrity.

CIAKI is a serious iatrogenic complication in clinical practice, but its mechanism has not been fully elucidated due to its complex pathophysiology. Previous studies have focused on the toxic effect of CM on renal tubules, but some studies suggest that renal vascular damage caused by CM and toxic effects on renal tubular cells are the key factors in CIAKI [12,33]. PTCs, which are the main vessels supplying the renal tubules and interstitium, consist of small straight vessels and cortical PTCs [34,35]. The small straight medullary vessels are essential for regulating renal medullary blood flow, and renal medullary hypoxia is central to the pathophysiology of CIAKI [36]. Experimental studies have indicated that vascular injury, characterized by impaired endothelial cell function, is a hallmark of CIAKI [12]. When CM enter the body through an artery or vein and directly interacts with vascular endothelial cells, their cytotoxicity can lead to apoptosis or necrosis of these cells, as demonstrated in both in vitro and in vivo studies [36,37]. CM exposure increases mitochondrial dysfunction and oxygen consumption, resulting in the accumulation of ROS. This ROS accumulation induces endothelial cell apoptosis and inflammation, exacerbating endothelial injury and further contributing to mitochondrial renal dysfunction and parenchymal hypoxia due to endothelial dysfunction and renal tubular transport disorders [38,39]. Additionally, CM inhibit the production of vasodilators (such as nitric oxide and prostacyclin) while promoting the production of vasoconstrictors (like endothelin) in endothelial cells, leading to PTC vasoconstriction and renal ischemia [40]. Endothelial dysfunction following CM administration also diminishes the anti-inflammatory and antithrombotic properties of blood vessels, increasing the risk of systemic and organ-specific complications [39]. Elevated levels of plasma endothelial cell markers (von Willebrand Factor and tissue-type plasminogen activator) have been observed after CM injection in rats, and CM have been shown to induce the release of circulating endothelial microparticles in patients with cardiovascular disease after angiography [41]. Therefore, improving endothelial dysfunction in PTCs may represent a viable strategy for preventing CIAKI. Endothelial barrier function is regulated by intercellular AJs and TJs [13,42]. VE-cadherin, the principal component of intercellular adhesion, interacts with the actin cytoskeleton via associated proteins such as α-catenin and β-catenin. The VE-cadherin-catenin complex is dynamic and disappears from the extracellular space in response to various stimuli, such as thrombin, that reduce endothelial barrier function [43].

IMD, a member of the CGRP family, shares 28% homology with adrenomedullin [18]. IMD plays a crucial role in regulating homeostasis and maintaining various physiological and pathological functions. It exerts multiple biological effects, including vasodilation, stabilization of the endothelial barrier, and inhibition of cell apoptosis. Similar to adrenomedullin, IMD enhances the production of intracellular cAMP across various systems, including the cardiovascular system, through the calcitonin receptor-like receptor/receptor activity-modifying protein (CLR/RAMP) receptor complexes [44,45]. It is recognized that cAMP is one of the most effective signaling molecules for stabilizing the endothelial cell barrier. The two main signaling pathways downstream of cAMP are the activation of PKA and the exchange protein directly activated by cAMP [46]. These pathways activate guanine nucleotide exchange factors (GEFs), such as Tiam1 and Vav2, thereby stabilizing the endothelial barrier with AJs mediated by Rac1 and improving the cortical actin cytoskeleton. In this study, we demonstrated that IMD, in vitro, directly increased cAMP levels, elevated Rac1 levels, activated the cAMP/Rac1 pathway, and mitigated iohexol-induced damage to adhesion and connections between HUVECs. Additionally, IMD restored HUVEC viability, which was compromised by iohexol, and attenuated iohexol-induced HUVEC apoptosis. We further validated our findings using a rat CIAKI model, where IMD treatment resulted in increased Rac1 and VE-cadherin expression in the kidneys of CIAKI rats. Compared to the CIAKI group, IMD-treated CIAKI rats exhibited significantly enhanced renal CD34-positive staining and reduced renal tubular injury scores. Transmission electron microscopy revealed that IMD significantly mitigated the destruction of the PTC endothelial barrier compared to the CIAKI group; however, this protective effect was abolished when Rac1 was blocked by NSC23766. These results indicate that IMD protects intercellular junctions in PTCs, stabilizes the endothelial barrier, and reduces CIAKI through activation of the cAMP/Rac1 pathway. IMD has promising clinical application prospects across various pathological conditions. This includes its role in the tumor microenvironment and its application in clinical studies of anti-tumor drugs [47]. Additionally, IMD shows potential for use in the treatment of sepsis and renal ischemia-reperfusion injury [48]. Studies have demonstrated that intermedin alleviates renal ischemia-reperfusion injury and enhances neovascularization in Wistar rats [29]. Future research should further explore the therapeutic potential of IMD in clinical applications.

There are some limitations to this study. We utilized HUVECs instead of RPECs, which may not fully simulate the physiological state of RPECs. We plan to validate our findings in future studies using RPECs or other kidney-specific endothelial cell models to further enhance the translational relevance of our results. Additionally, IMD can bind to and activate various G proteins, exerting a range of biological effects through different signaling pathways. Consequently, we cannot entirely rule out the possibility that IMD may play a protective role in the kidney through other mechanisms. In conclusion, our findings demonstrate that IMD protects the endothelial junctions of PTCs, stabilizes the endothelial barrier, and preserves renal function via the cAMP/Rac1 pathway in the CIAKI model. Therefore, we propose that IMD holds potential as a novel preventive agent for CIAKI, and its clinical significance warrants further investigation.

## 4. Materials and Methods

### 4.1. Reagents and Antibodies

The following primary antibodies were used for immunoblotting or immunofluorescence: VE-cadherin (361900; ThermoFisher Scientific, Waltham, MA, USA); CD34 (BM4082, Boster, Wuhan, China); ICAM1 VEGFR2 (A00901-3, Boster, Wuhan, China); Rac1/Cdc42 (4651, Cell Signaling Technology, Danvers, MA, USA); Phospho-Rac1/cdc42 (#2461, Cell Signaling Technology, Danvers, MA, USA); PKA alpha + beta (GB11598; ServiceBio, Wuhan, China); and GAPDH (BM3874, Boster, Wuhan, China). Secondary antibodies were HRP conjugated AffiniPure goat anti-rabbit IgG (H + L) (BA1054; Boster, Wuhan, China); IMD1-53(010-60; Phoenix Pharmaceuticals, Burlingame, CA, USA); NSC23766 (HY-15723A MedChemExpress, Monmouth Junction, NJ, USA); iohexol (Omnipaque, 350 mg I/mL, GE Healthcare Company, Shanghai, China), osmotic minipumps (ALZET model 1003; DURECT Corporation, Cupertino, CA, USA); Creatinine (Cr) Assay Kit (C011-2-1; NJJC BioInstitute, Nanjing, China); cAMP kit (RXJ99870; Ruixin Biotech; Quanzhou, China); Cell Counting Kit-8 (HY-K0301, MedChem Express, Monmouth Junction, NJ, USA); FITC Annexin V Apoptosis Detection Kit I (#556547, BD Biosciences, Franklin Lakes, NJ, USA); and DMEM (Gibco Co., Carlsbad, CA, USA). All other chemicals and reagents were of analytical grade and were obtained from commercial sources.

### 4.2. Cell Culture

HUVECs (Cat No.FH1122) were provided by Shanghai Fuheng Biotechnology Co., Ltd. (Shanghai, China). The cells were maintained at 37 °C in a humidified atmosphere with 5% CO_2_. Once the cells reached 80–90% confluence, they were detached using 0.25% trypsin-EDTA and subcultured at a dilution of 1:3 or 1:4 into new 100 mm culture dishes. To ensure the cellular vitality and stability of the phenotype, all experiments utilized HUVECs at passage numbers between 3 and 5. They were serum-deprived with 0.5% fetal bovine serum (FBS)-ECM for 6 h before further treatment. 

### 4.3. Cell Viability Assay

HUVECs were preincubated with IMD (0, 1, 10, or 100 nmol/L) for 30 min and subsequently treated with iohexol (10, 20, 40 or 80 mg I/mL) for 12 h. Cell viability was evaluated using the Cell Counting Kit-8 (CCK-8). All experiments were repeated three or four times.

### 4.4. Apoptosis Assay

Apoptosis was assayed using flow cytometry analysis. HUVECs were pretreated with or without IMD (10 nmol/L) for 30 min and then incubated with iohexol (40 mg I/mL) or iohexol + NSC23766 (50μM) for 12 h. Cell apoptosis was tested using an Annexin V-FITC apoptosis detection kit. Briefly, HUVECs were washed with PBS, resuspended in Annexin V binding buffer, and incubated with Annexin V-FITC and propidium iodide for 15 min at room temperature in the dark. The cells were then analyzed by flow cytometry (Beckman Coulter, Brea, CA, USA) within 1 h. All experiments were repeated three to four times.

### 4.5. ELISA

All cAMP levels were determined by ELISA, according to the manufacturer’s instructions using the cAMP kit (RXJ99870; Ruixin Biotech; Quanzhou, China).

### 4.6. Immunoblotting

Homogenized renal tissue and cell lysate were separated onto 8% or 10% SDS-PAGE gels, transferred to polyvinylidene difluoride membranes, and then probed with the following antibodies: GAPDH, Rac1, Phospho-Rac1, VE-Cadherin, and VEGFR2. All antibodies were diluted in 3% BSA.

### 4.7. Animals, CIAKI Model, and Treatments

Male Sprague–Dawley (SD) rats, 6–8 weeks old and weighing 180–210 g, were purchased from the Shanxi Medical University Experimental Animal Center (Taiyuan, China) and maintained in a specific pathogen-free environment at our facility. All animals were fed standard food and had free access to water. All animal experiments were conducted humanely and according to the Institutional Animal Care Instructions. The animal study was approved by the Ethics Committee of the Second Hospital of Shanxi Medical University (IACUC Agreement No: DW2022012).

A CIAKI model was reproduced as previously described in detail with minor modifications [49]. Rats were dehydrated for 48 h and were administered an intramuscular injection of furosemide (10 mL/kg; Harvest Pharmaceutical, Shanghai, China) 30 min before the intravenous injection of iohexol (15 mL/kg; Omnipaque, 350 mg I/mL, GE Healthcare Company, Shanghai, China) through the great saphenous vein.

The rats were randomly divided into the following five groups: Sham, IMD + Saline, CIAKI, and CIAKI + IMD. IMD was infused at a dose of 300 ng/kg/h using a subcutaneously implanted Alzet osmotic minipump 24 h before administration of iohexol; CIAKI + IMD + NSC23766: NSC23766 (8 mg/kg) was injected intraperitoneally 30 min before iohexol administration. Rats were euthanized with an overdose of sodium pentobarbital (150 mg/kg body weight) administered by intraperitoneal injection and sacrificed after 24 h. Blood and kidneys were harvested for further analysis.

### 4.8. Biochemical Evaluation of Renal Function

Serum creatinine levels were determined using enzyme methods with a Creatinine (Cr) Assay Kit (C011-2-1; Nanjing Jiancheng Bioengineering Institute, Nanjing, China).

### 4.9. Histopathological Examination

Kidney tissue was fixed in 10% formalin for 24 h at 4 °C and embedded in paraffin blocks. Sections were cut at 2.5–3 μm and stained with hematoxylin and eosin (HE) and periodic acid Schiff (PAS) reagent, according to the standard routing protocols. 

Renal tubular damage was classified on six levels based on the loss of the brush border, tubular dilation, cast formation, tubular necrosis, and neutrophil infiltration. Ten high-power fields (original magnification ×400) were chosen randomly, and each field was scored from 0 to 5 (0: normal; 1: mild injury, involvement of 0–10%; 2: moderate injury, involvement of 11–25%; 3: severe injury, involvement of 26–49%; 4: high severe injury, involvement of 50%–75%; 5: extensive injury, involvement of >75%). All evaluations were performed by two investigators blinded to experimental conditions.

### 4.10. Immunohistochemistry and Immunofluorescence

Immunohistochemistry and immunofluorescence staining were performed on paraffin-embedded slices as previously described. The antibodies used in this study were cd34 (BM4082, Boster, Wuhan, China) and ICAM1 (A00171, Boster, Wuhan, China).

### 4.11. Transmission Electron Microscope Analysis

Tissue samples were prefixed with 3% glutaraldehyde, then the tissue was postfixed in 1% osmium tetroxide, dehydrated in series acetone, extensively infiltrated, and embedded in Epox 812. The semi-thin sections were stained with methylene blue, and ultra-thin sections were cut with a diamond knife, stained with uranyl acetate and lead citrate. Sections were examined with a transmission electron microscope (JEM-1400-FLASH, JEOL Ltd., Tokyo, Japan).

### 4.12. Statistical Analyses

Continuous data were reported as the mean ± standard deviation (SD). Student’s T-test or ANOVA were performed to detect significance between groups if the data were normally distributed, while the Mann–Whitney *U* test or Kruskal–Wallis test was used if the data were non-normally distributed. To assess subgroup analysis, the Tukey test or Dunn’s multiple comparisons test was used. All statistical analyses and graphs were obtained using SPSS v22.0 (IBM, New York, NY, USA) and GraphPad Prism 9.3.1 (La Jolla, CA, USA) software. A two-tailed *p*–value of <0.05 was considered to indicate a statistically significant difference.

## Data Availability

Data are contained within the article.
